# Increased robustness of early embryogenesis through collective decision-making by key transcription factors

**DOI:** 10.1186/s12918-015-0169-8

**Published:** 2015-06-02

**Authors:** Ali Sharifi-Zarchi, Mehdi Totonchi, Keynoush Khaloughi, Razieh Karamzadeh, Marcos J. Araúzo-Bravo, Hossein Baharvand, Ruzbeh Tusserkani, Hamid Pezeshk, Hamidreza Chitsaz, Mehdi Sadeghi

**Affiliations:** Department of Bioinformatics, Institute of Biochemistry and Biophysics, University of Tehran, Tehran, Iran; Department of Stem Cells and Developmental Biology at Cell Science Research Center, Royan Institute for Stem Cell Biology and Technology, ACECR, Tehran, Iran; Department of Genetics at Reproductive Biomedicine Research Center, Royan Institute for Reproductive Biomedicine, ACECR, Tehran, Iran; Department of Biophysics, Institute of Biochemistry and Biophysics, University of Tehran, Tehran, Iran; Computational Biology and Bioinformatics Group, Max Planck Institute for Molecular Biomedicine, Münster, Germany; Group of Computational Biology and Systems Biomedicine, Biodonostia Health Research Institute, 20014 San Sebastián, Spain; IKERBASQUE, Basque Foundation for Science, 48011 Bilbao, Spain; School of Computer Science, Institute for Research in Fundamental Sciences, Tehran, Iran; School of Mathematics, Statistics and Computer Sciences, Center of Excellence in Biomathematics, College of Science, University of Tehran, Tehran, Iran; School of Biological Science, Institute for Research in Fundamental Sciences (IPM), Tehran, Iran; Computer Science Department, Colorado State University, Fort Collins, Colorado 80523 USA; National Institute of Genetic Engineering and Biotechnology (NIGEB), Tehran, Iran

**Keywords:** Waddington landscape, Early embryogenesis, Differentiation, Developmental bifurcations, Genetic circuit, Single cell analysis

## Abstract

**Background:**

Understanding the mechanisms by which hundreds of diverse cell types develop from a single mammalian zygote has been a central challenge of developmental biology. Conrad H. Waddington, in his metaphoric “epigenetic landscape” visualized the early embryogenesis as a hierarchy of lineage bifurcations. In each bifurcation, a single progenitor cell type produces two different cell lineages. The tristable dynamical systems are used to model the lineage bifurcations. It is also shown that a genetic circuit consisting of two auto-activating transcription factors (TFs) with cross inhibitions can form a tristable dynamical system.

**Results:**

We used gene expression profiles of pre-implantation mouse embryos at the single cell resolution to visualize the Waddington landscape of the early embryogenesis. For each lineage bifurcation we identified two clusters of TFs – rather than two single TFs as previously proposed – that had opposite expression patterns between the pair of bifurcated cell types. The regulatory circuitry among each pair of TF clusters resembled a genetic circuit of a pair of single TFs; it consisted of positive feedbacks among the TFs of the same cluster, and negative interactions among the members of the opposite clusters. Our analyses indicated that the tristable dynamical system of the two-cluster regulatory circuitry is more robust than the genetic circuit of two single TFs.

**Conclusions:**

We propose that a modular hierarchy of regulatory circuits, each consisting of two mutually inhibiting and auto-activating TF clusters, can form hierarchical lineage bifurcations with improved safeguarding of critical early embryogenesis against biological perturbations. Furthermore, our computationally fast framework for modeling and visualizing the epigenetic landscape can be used to obtain insights from experimental data of development at the single cell resolution.

**Electronic supplementary material:**

The online version of this article (doi:10.1186/s12918-015-0169-8) contains supplementary material, which is available to authorized users.

## Background

**Fig. 1:**
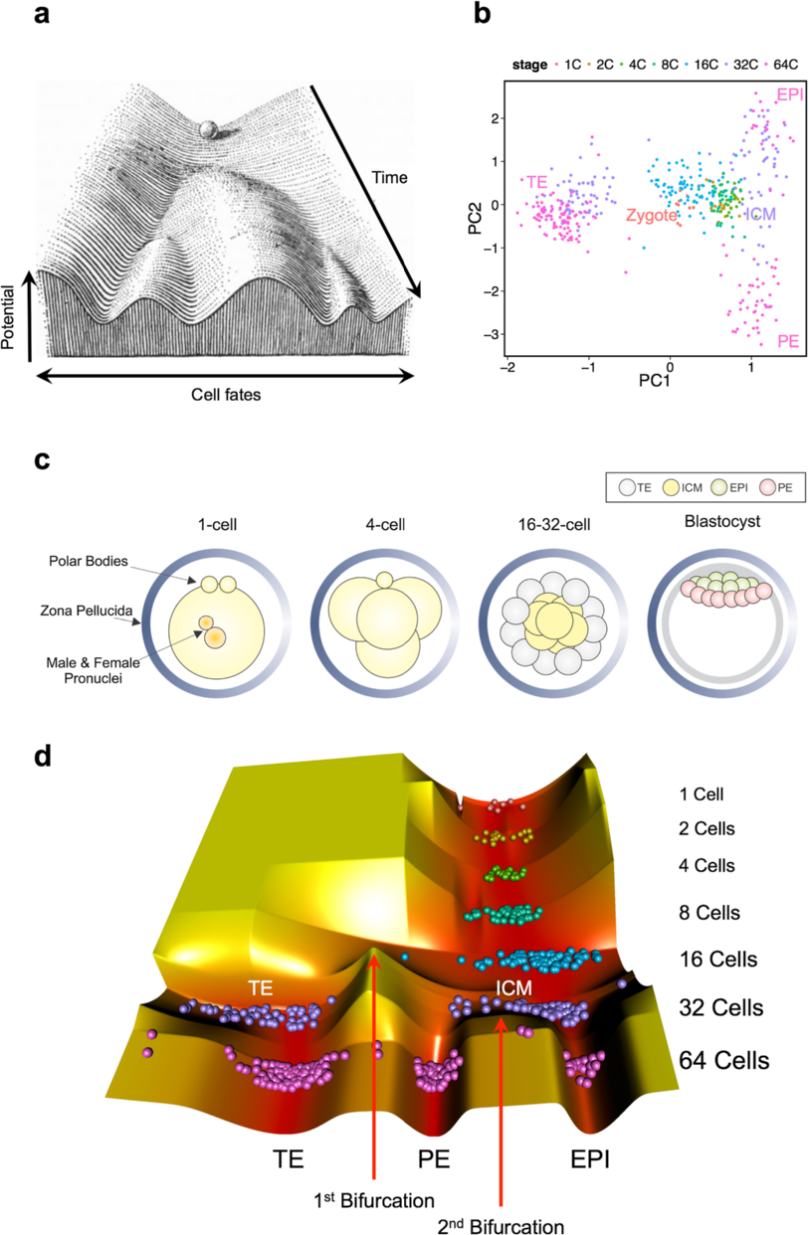
Waddington landscape of the mouse preimplantation embryo. **a** The original artwork of Waddington (we have added the arrows and the labels). **b** Principal component analysis (PCA) of the mouse preimplantation embryo gene expression profiles. Each point represents one cell, and the color of each point shows the developmental stage of the cell. **c** Schematic representation of mouse preimplantation embryonic development. **d** The computational Waddington landscape of the mouse early development based on the gene expression profiles. Each ball represents a single cell. PC: Principal component, ICM: Inner cell mass, TE: Trophectoderm, PE: Primitive endoderm, EPI: Epiblast

More than six decades ago, Conrad H. Waddington portrayed a conceptual landscape of development (Fig. 1a). In his “epigenetic landscape” a ball that indicates the whole or part of an egg or an embryo is rolling down a sloping and undulating surface with several valleys that represent distinguished organs or tissues [1]. Beyond its deceptive simplicity, the epigenetic landscape has entailed numerous embryogenesis facts: (i) decreased differentiation potency during development as illustrated by tilt of the landscape; (ii) the epigenetic barriers between sharply distinct cell fates, depicted as the hills between the valleys; (iii) derivation of distinct cell types from identical cells, portrayed as bifurcated valleys.

Waddington’s innovation suggested that genetic interactions were the major determinants of a landscape’s shape [1, 2]. In support of this idea, a genetic circuit of two TFs each stimulating itself (auto-activation) and repressing the activity of the other (mutual inhibition) has been shown to form a tristable dynamical system [3]. This system can model a lineage bifurcation, which is the differentiation of two distinct cell types from the common progenitor. The triple stable steady states or “attractors” represent the progenitor and two bifurcated lineages. In the progenitor cell state both TFs are expressed at balanced rates. In either of two bifurcated cell states, one TF is active or highly expressed whereas the other TF is silent or slightly expressed.

An example of the mutual-inhibition and auto-activation circuit between two TFs is the *Gata1* versus *Pu.1* circuit, which has been proposed to govern the bifurcation of common myeloid progenitors (*Gata1*+/*Pu.1*+) to either erythroids (*Gata1*+/*Pu.1*-) or myeloids (*Gata1*-/*Pu.1*+) [3]. Other examples of two-TF regulatory circuits suggested for lineage bifurcations are provided in Table 1. Furthermore, a hierarchy of mutual-inhibition and auto-activation circuits among several pairs of TFs is suggested for the hierarchy of cell type bifurcations during early development [4, 5] and pancreatic differentiation [6].

**Table 1:** Examples of two-TF regulatory circuits that are suggested for lineage bifurcations

TF1	TF2	Progenitor	Lineage 1	Lineage 2	Ref.
		(TF1 ≈ TF2)	(TF1 > TF2)	(TF1 < TF2)	
*Gata1*	*Pu.1*	Common myeloid progenitor	Erythroid	Myeloid	[3]
*Oct4*	*Cdx2*	Totipotent embryonic cells	Inner cell mass	Trophectoderm	[12]
*Nanog*	*Gata4/6*	Inner cell mass	Epiblast	Primitive endoderm	[13]
*Sox10*	*Phox2b*	Bipotential neural progenitor	Glia	Neuron	[54]
*Ptf1a*	*Nkx6*	Pancreatic progenitor	Exocrine cells	Endocrine cells	[55]
*Pax3*	*Foxc2*	Dermomyotome progenitor	Myogenic cells	Vascular cells	[55]

As a major drawback, the two-TF circuit is highly dependent on the concentrations and functions of a pair of TFs. In this model, a genetic or environmental perturbation that affects one of the TFs can change the behavior of the circuit and result in a deficient lineage bifurcation. Some experimental studies, however, show the cell differentiation is more robust.

For instance, the recent finding that the inner cell mass (ICM) is formed after complete inactivation of *Oct4* expression [7] rejects the hypothesis that ICM vs. trophectoderm (TE) bifurcation is switched solely by the *Oct4* versus *Cdx2* circuitry.

Here we introduce a computational framework for modeling the epigenetic landscape. Using the single cell resolution gene expression profiles of preimplantation mouse embryonic cells [8] we visualize the Waddington landscape of early development. After analysis of the expression patterns of the key TFs that are suggested to form early lineage bifurcations, we provide an extended form of hierarchical regulatory circuitry in which each bifurcation is decided by two clusters of TFs, rather than two single TFs. We show this extended circuitry is more robust against perturbation, which suggests it can better safeguard the development.

## Results

### The Waddington landscape of a preimplantation embryo

We constructed the epigenetic landscape of mouse preimplantation embryonic development using the expression profiles of 48 genes – mostly TFs – in 442 single pre-implantation embryonic cells [8]. For this purpose, we quantified three axes: cell type (x-axis), time of development (y-axis), and pseudo-potential function (z-axis, see methods for more details). Time of development was quantified according to the developmental stage of each cell in the dataset. We used principal component analysis (PCA) [9] to project the expression profiles of the cells into a two-dimensional space (Fig. 1b), in which the cells with similar fates during embryonic development (Fig. 1c) were clustered together. The angular coordinates of the cells in the PCA plot were used to put them across the x-axis of the epigenetic landscape. In this way the cells were sorted along the x-axis according to their types. We also defined a pseudo-potential function using the Gaussian mixture model and Boltzmann distribution, and computed the z-coordinates accordingly.

The result is shown in Fig. 1d. Each ball represents a single embryonic cell. The y-axis (back-to-front) shows different developmental stages from 1-cell (zygote) to 64-cell (blastocyst). The height of each region shows the pseudo-potential function level, which reflects both stability and differentiation potency. There is a single valley from the 1- to 16-cell stages that shows no significant difference between single embryonic cells at these stages. The first bifurcation appears at the 32-cell stage, where ICM is distinguished from TE. At the 64-cell stage the ICM cells undergo a second bifurcation that discriminates epiblast (EPI) from primitive endoderm (PE).

### Regulatory circuitry of two transcription factors (TFs) can form lineage bifurcations

**Fig. 2:**
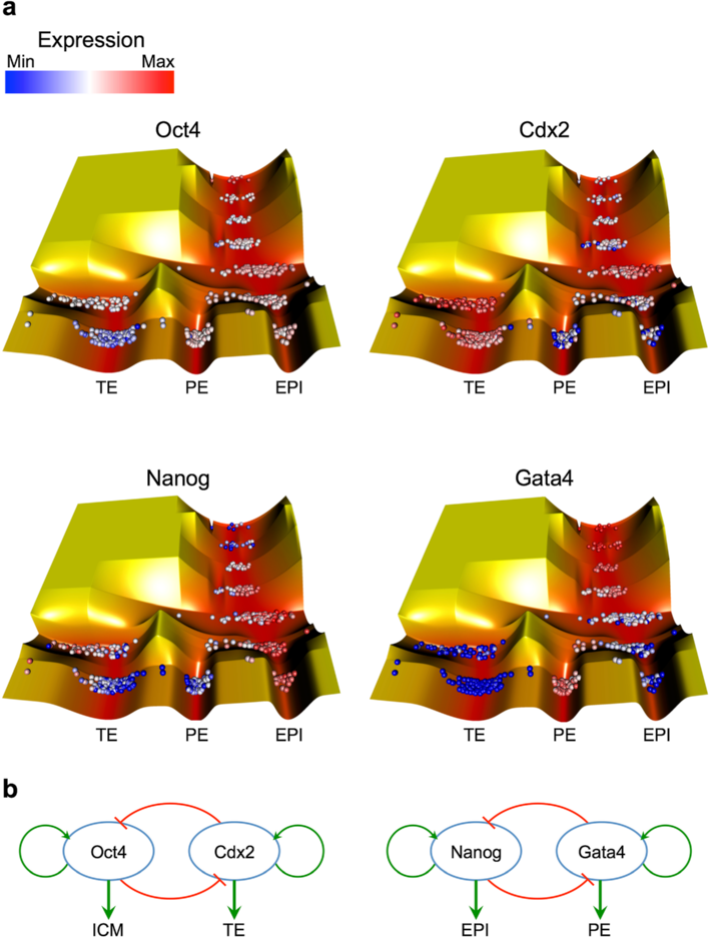
Expression levels of four key transcription factors (TFs) in early embryogenesis. **a** The gene expression levels of *Oct4*, *Cdx2*, *Nanog* and *Gata4* in the single cells of preimplantation embryos. The cells with the highest expression level of each TF are depicted in red, while the intermediate and the lowest expression levels are shown as white and blue, respectively. **b** The regulatory circuitry between *Oct4* and *Cdx2* (left)*,* and *Nanog* and *Gata4* (right). Green and red arrows show positive and negative regulatory interactions, respectively. TE: Trophectoderm, PE: Primitive endoderm, EPI: Epiblast

In order to inspect how the epigenetic landscape bifurcations were formed we examined the expression levels of four key TFs of preimplantation development: *Oct4*, *Cdx2*, *Nanog* and *Gata4*. These TFs were selected due to their known critical functions in the formation of early embryonic cell types [10, 11]. Our analysis shows that *Oct4* is expressed in ICM and its sub-lineages, but becomes silent in the TE valley (Fig. 2a). In contrast, *Cdx2* is overexpressed in the TE, and underexpressed in the ICM and its sub-lineages. Both *Nanog* and *Gata4* are underexpressed in the TE valley, but have a sharp contrast in ICM sub-lineages. *Nanog* is overexpressed in the EPI and underexpressed in the PE cells, while *Gata4* is overexpressed in the PE and underexpressed in the EPI valley.

Competition in expression of *Oct4* and *Cdx2* is suggested to arise from the particular form of regulatory circuitry between them [12]. While binding of *Oct4* to its own promoter has a positive regulatory effect, its binding to the *Cdx2* promoter is suppressive. Similarly, *Cdx2* activates itself but inhibits *Oct4* (Fig. 2b, left). The regulatory circuitry between *Nanog* and *Gata4/6* has a similar structure (Fig. 2b, right) [13, 14].

**Fig. 3:**
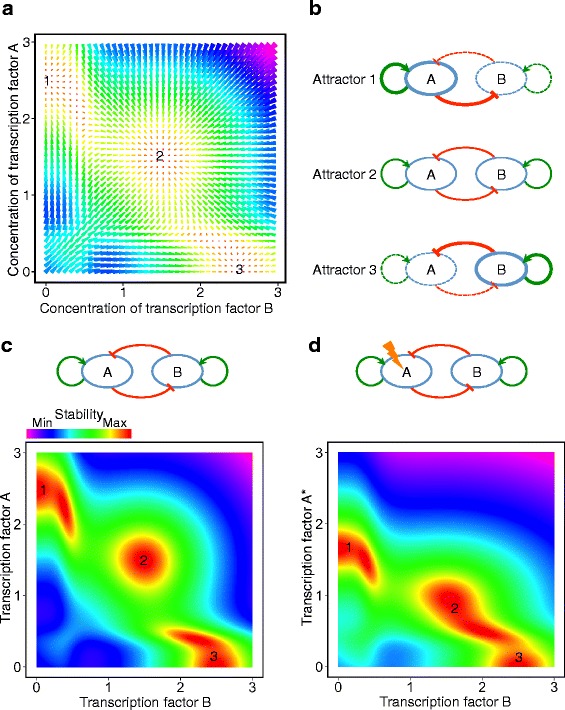
Attractor states of the two-TF regulatory circuitry. **a** Force-field representation of the dynamical system of a regulatory circuitry consisting of two TFs with auto-activation and mutual-repression interactions. **b** Regulatory states of the TFs in the three enumerated attractor states. Highly expressed TFs and strong interactions are shown as thick lines, whereas thin lines represent intermediate expressions or interactions. Null expressions or interactions are depicted as dashed-lines. **c**, **d** Phase space representations of the two-TF circuits. Red regions represent the highly stable states. (c) Both TFs have equal degradation rates. **d** The degradation rate of the transcription factor A is increased by 50 % (denoted by A*)

A set of ordinary differential equations (ODEs) are previously used to model the regulatory circuitry between two generic TFs, such as A and B, with auto-activation and mutual inhibitions [12] (see Methods section for more details). Such ODEs form a tristable dynamical system that can be visualized in a force-field representation (Fig. 3a). Each grid point of the plot represents one system state with certain concentration levels of the TFs A and B, which are specified as the point dimensions. For each grid point, an arrow shows the direction of changes in the TF concentrations after a short period of time. The areas with longer arrows, in violet, represent the system states with higher tendency to change. In contrast, the shorter red arrows represent the more stable states of the system.

In the attractor 1, as enumerated in Fig. 3a, A is highly expressed and B is silent, and this state is maintained through the positive and negative feedback loops (Fig. 3b, top). The same conditions hold for the attractor 3 in which dominant expression of B suppresses expression of A and maintains a high abundance of B (Fig. 3b, bottom). In attractor 2, however, both TFs are expressed at lower and balanced rates (Fig. 3b, middle). In the same attractor, the positive feedback each TF receives from auto-activation forms equilibrium with the negative feedback from the other TF. The attractor 2 represents a progenitor cell type, while 1 and 3 denote two bifurcated cell lineages.

### Two-cluster regulatory circuitry can resist perturbations

Although the two-TF regulatory circuitry could account for a developmental bifurcation, we conjectured that this type of regulatory circuitry would be too sensitive. In other words, genetic mutations or environmental perturbations that affect the concentration or function of either TF could influence the bifurcation and the ratios of the cells that differentiate into either lineage, or even lead some cell type to completely vanish.

To test this conjecture, we computationally examined the effect of an increased degradation rate of one TF. As shown in Fig. 3c, the original two-TF circuit with similar degradation rates of both TFs forms three attractor states indicated by red areas surrounded by the green epigenetic barriers. Increasing the degradation rate of the protein A by 50 % in the ODE model significantly changes the position of the stable states (Fig. 3d, the more degradable form of protein A is denoted by A*). While the attractor 1 remains isolated, the attractors 2 and 3 fuse together. As a result, it would be more likely for the progenitor cells in attractor 2 to differentiate into the attractor 3 rather than 1 during the lineage bifurcation.

**Fig. 4:**
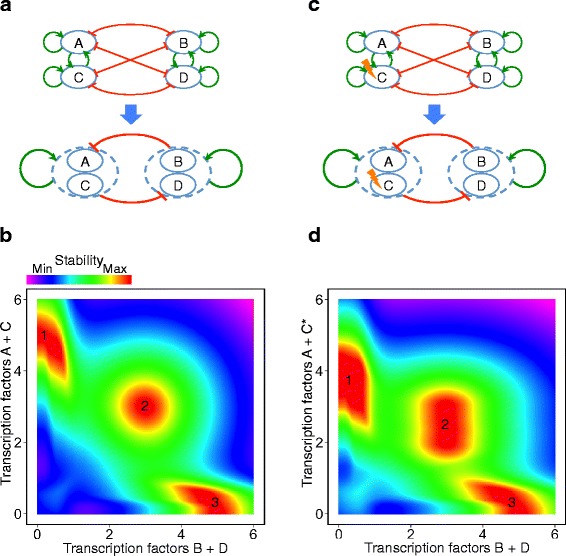
Attractor states of the two-clusters regulatory circuitry. **a** The regulatory circuitry consisting of two clusters: A and C in one cluster, and B and D in the other. The interactions between the members of the same cluster are positive, and the interactions between the TFs of different clusters are negative. **b** Phase space representation of the system. Red regions are highly stable. **c**, **d** Regulatory circuitry and phase space representation of two clusters, in which the degradation rate of the protein C is increased by 50 % (denoted as C*)

We hypothesized that the regulatory circuitry would be more robust against perturbations or noise if there were more TFs involved in the formation of either branch of the bifurcation. To check this hypothesis we designed a new ODE system that represented a regulatory circuitry consisting of two clusters, with a couple of TFs in each cluster. The TFs of the same cluster have positive mutual regulatory interactions, whereas the TFs of opposite clusters inhibit each other (Fig. 4a).

To show a 4-dimentinal (4D) expression-space of the 4 TFs as a 2D plot, we assigned the total expression of the TFs in each cluster to one axis (Fig. 4b). The pseudo-potential function of the two-TF cluster circuitry shows a tristable system, which is very similar to the two-TF model. Both TFs A and C that belong to the same cluster are highly expressed in the attractor 1, whereas B and C are silent. In contrast, B and D are overexpressed in the attractor 3, while A and C are silent. The progenitor attractor state 2 represents the equilibrium in which all TFs are expressed at balanced rates.

In the two-cluster circuit, we analyzed the effect of a 50 % increase in the degradation rate of protein C (Fig. 4c, d). The attractor areas are slightly moved in the perturbed model (Fig. 4d) compared to the original two-cluster model (Fig. 4b). In particular, attractor 2 is slightly closer to attractor 3, due to the decreased concentration of protein C in the equilibrium state. However all three attractors are maintained and none them are fused together.

To have a quantitative insight into the robustness, we simulated the differentiation of four cell populations, each population having one of the regulatory circuitries shown in Fig. 3c, d and Fig. 4a, c (see the Methods section and the Additional file 1 for more details). We forced the cells to leave the progenitor state (the attractor 2 in Figs. 3 and 4) and differentiate into the attractor states 1 or 3. This was performed by gradually decreasing the auto-activation strengths of the TFs, as previously suggested [15].

In both two-TF and two-cluster circuits, the number of cells that differentiate into the attractors 1 and 3 are very similar (maximum 1 % difference), when there is no perturbation. After increasing degradation rate of one TF, only 3 % of the cells with two-TF circuit differentiate to the attractor 1. Nevertheless, the fraction of the cells with two-cluster circuit that differentiate to the attractor 1 is significantly higher (24 %). This simulation shows that one cell lineage (attractor 1) is almost vanished when the two-TF circuit is perturbed, while the two-cluster circuit is significantly more robust and safeguards differentiation into both lineages.

### Early developmental bifurcations are switched by two clusters of TFs

**Fig. 5:**
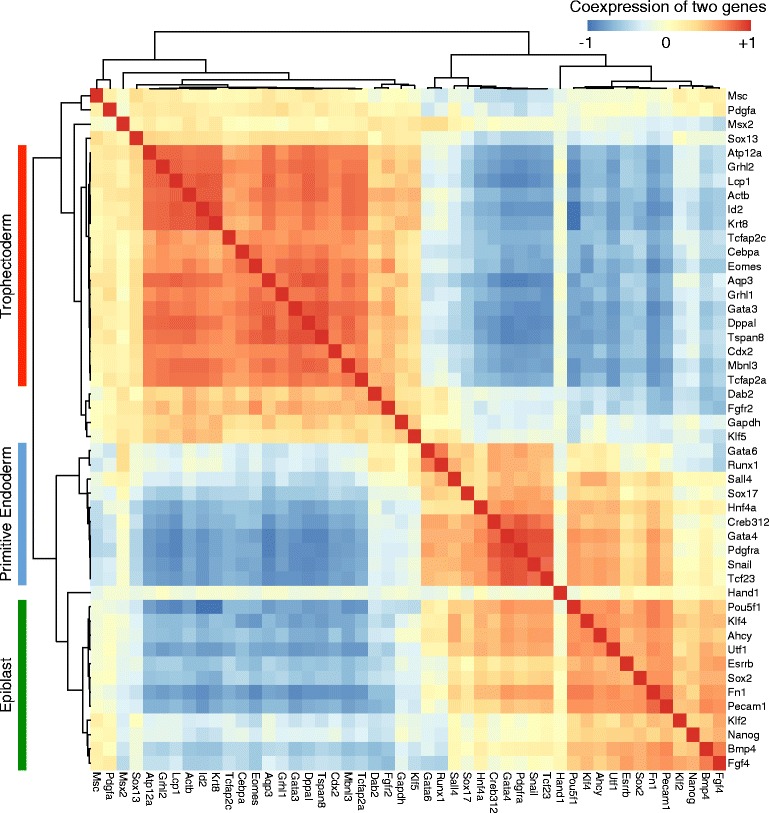
Co-expressions of 48 genes in single blastocysts of the 64-cell stage mouse embryos. Each square shows the correlation value between expression profiles of two genes. Hierarchical clustering trees of the genes are shown in the top and left sides. There are three clusters of genes with high positive correlations, as indicated on the left side. The cell types in which each cluster is highly expressed are also shown

We sought to determine whether the hypothesized TF clusters existed in the regulatory circuitry of the early embryogenesis. For this reason, we analyzed the expression profiles of the single mouse blastomeres at the 64-cell stage (Fig. 1b, c). Our analysis indicates three clusters of genes, which are mostly TFs (Fig. 5). The expression profiles of the genes in the same cluster are highly correlated, but lower or negative correlations are observed among the genes of different clusters. The first cluster consists of 17 genes, including *Cdx2*, *Eomes* and *Gata3,* which are highly expressed in TE. The second cluster includes 10 genes such as *Gata4*, *Gata6* and *Sox17* that mark PE cells. The 12 genes of the third cluster, including *Nanog*, *Fgf4* and *Sox2,* are overexpressed in EPI cells. The genes of the TE cluster show lower coexpression with the genes of the other clusters. Some EPI genes are highly coexpressed with PE genes, which might reflect the limited time passed from the bifurcation of EPI and PE cell types at 64-cell stage.

**Fig. 6:**
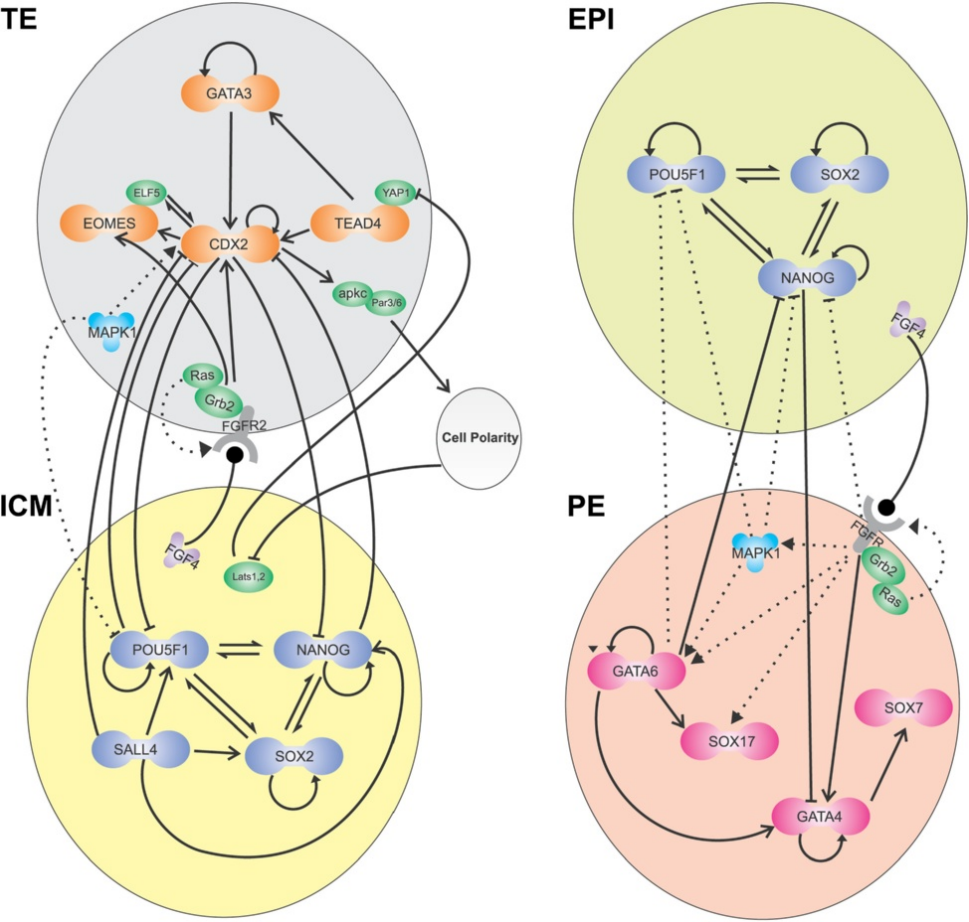
Regulatory circuitry of lineage bifurcations in the mouse preimplantation embryo. Left side shows two clusters of genes that are active either in the ICM or the TE. The interactions among the genes of each cluster are positive, while the interactions between the members of distinct clusters are negative. Right side shows similar network for the EPI and the PE. ICM: inner cell mass, TE: trophectoderm, EPI: epiblast, PE: primitive endoderm

Through a literature search we revealed the experimentally validated regulatory interactions among the genes that pioneer early lineage bifurcations [8, 13, 16–27]. There are reports of positive interactions among *Tead4*, *Eomes, Gata3*, *Cdx2*, *Elf5* and a number of other genes that are upregulated in TE cells (Fig. 6). The regulatory effects among *Pou5f1*(Oct4), *Nanog*, *Sox2* and *Sall4*, as key TFs of the ICM cells, are also positive. However, the TFs in one cluster have been shown to repress the TFs in the other cluster. This finding is in agreement with the structure of the two-cluster circuitry. A similar regulator pattern can also be observed among the PE markers *Gata4*, *Gata6*, *Sox17* and *Sox7* in one cluster, and EPI markers *Nanog*, *Sox2* and *Oct4* in the other cluster. Assigning the color of the cells on the epigenetic landscape based on the average expression level of each cluster confirmed the proposed TF clusters experimentally (see the Additional file 2).

## Discussion

We computationally visualized the Waddington landscape of mouse preimplantation development using the experimental data and depicted the differentiation of cell lineages as bifurcations of the valleys. In this study, we modeled the dynamical system of a regulatory circuit consisting of two individual TFs with auto-activation and mutual inhibitions, which has been proposed for lineage bifurcation [5, 15, 18]. This circuit formed a tristable dynamical system with clear borders of epigenetic barriers among them. An increased degradation rate of one TF caused the epigenetic barriers between the progenitor and one of the lineage committed cell states to be broken. This experiment showed that the circuit of two individual TFs is not very robust, and the ratios of the cells that commit to each lineage may be significantly affected by perturbations.

We investigated whether the presence of more TFs in the regulatory circuitry that governs a developmental bifurcation could lead to a more robust system. Extension of the initial circuit to a pair of clusters with multiple lineage-instructive TFs in each cluster, which activated themselves and inhibited the other cluster members, resulted in another tristable dynamical, similar to the one formed by the two-TF circuit. In the extended network, however, the epigenetic barriers were not vastly affected by increased decay rate of one TF, which was quantitatively confirmed by a simulation.

The positive feedbacks from the other TFs of the same cluster could buffer the effect of perturbations on a particular TF. This buffering property is somehow similar to the Waddington’s original idea of “*canalisation”* – the capability of the system to recover after slight perturbations [1]. We expect this property would be even stronger in larger clusters of TFs having more positive feedback loops. This is in agreement with a suggestion by Waddington in the same book: “*canalisations are more likely to appear when there are many cross links between the various processes, that is to say when the rate of change of any one variable is affected by the concentrations of many of the other variables”* [1]. As the second property, the total expression of one TF cluster can overcome and inhibit the expression of the other TF cluster. We call these properties together as the collective decision-making of the TFs.

The extended regulatory circuitry was further illustrated by our analysis of the expression profiles of key TFs in mouse blastocysts. We indicated three clusters of genes (mostly TFs) that represented the EPI, PE and TE cell types (Fig. 5). A literature review of regulatory interactions among members of each cluster confirmed the structure of two-cluster regulatory circuitry and its role during early development (Fig. 6).

**Fig. 7:**
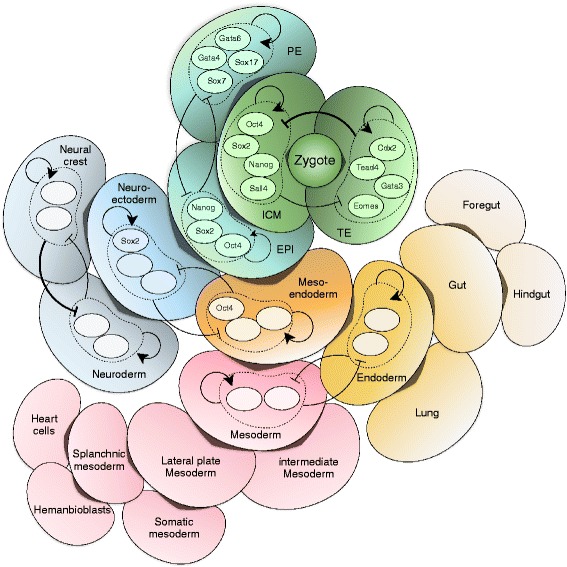
Developmental bifurcations are governed by a hierarchical regulatory circuitry. Each circuit consists of two clusters of transcription factors (TFs), with positive feedbacks within each cluster and negative feedbacks between the two clusters. Prior to each developmental bifurcation, the TFs of both corresponding clusters are expressed at a balanced state. In each post-bifurcation branch, one cluster is downregulated while the other is upregulated. This triggers the competitive expression of clusters that switch later bifurcations

The proposed concept of two-cluster circuitry can be extended in a modular way to form a hierarchy of developmental bifurcations (Fig. 7). Early stages of development involve minimal cell quantity, and a small change in the fate of each single cell will pass on to a large number of offspring cells. Thus stronger safeguarding against perturbations is more crucial in the early development. This can be achieved by the presence of more TFs in each cluster and/or stronger feedback loops. The later developmental bifurcations are less sensitive and might rely on smaller clusters or even individual TFs.

To identify the TF clusters of each bifurcation circuit we suggest assigning the expression profiles of embryonic and adult cell types to the network of differentiation [28]. Then we can look for the differentially expressed TFs and chromatin remodelers between a pair of cell types and offspring lineages, which are bifurcated from the common progenitor cells. This can be a systematic method to identify cocktails essential for cell type conversions such as reprogramming and transdifferentiation [29].

While the proposed hierarchical regulatory circuitry provides a basis for better understanding and analysis of developmental bifurcations, we do not exclude more complicated mechanisms such as the role of signaling networks and morphogens. For example, during embryonic stem cell differentiation, *Oct4* and *Sox2* have mutual positive feedbacks and belong to the same cluster of upregulated TFs in the ICM and EPI. The repressive effects of *Wnt3a* and activin on *Sox2,* and also inhibition of *Oct4* by *Fgf* and retinoic acid result in asymmetric upregulation of *Sox2* in the mesendoderm and *Oct4* in the neural ectoderm [30]. This example lends support to the concept that signaling cascade forces dominate regulatory interactions of TFs, and will eventually cause the TF cluster to split.

A second example of the cryptic mechanisms in bifurcation regulation is the presence of master and supportive TFs. In the symmetric computational model, we have assigned identical effects to different TFs of the same cluster in determining the cell lineage. This can be further extended to an asymmetric model where one, or a small number of TFs in each cluster are the master lineage indicators and the other members support their expression and function. The latter suggests inactivation of different TFs in the same cluster will have different effects on formation of the corresponding cell lineage, which is supported by experimental evidences [11].

There are even more aspects of the cell biology that are critical for understanding development and differentiation. While gene-to-gene interactions are essential for the cells to differentiate, cell-to-cell communications are crucial for the embryo to balance the required quantity of each cell type, and to develop tissues and organs. As an example, the ICM and EPI cells secrete the *Fgf4* signal, which binds to the *Fgfr2* receptor on the membrane of TE and PE cells (Figs. 5 and 6). The development of TE and PE cells are significantly influenced by this signal [31, 32]; for instance the increased *Fgf4* concentration results in enhanced PE and diminished EPI cells [33]. As a result, the proportion of the cells that differentiate into either EPI or PE would be balanced, which is another mechanism of developmental robustness. In absence of signals and intercellular communication, development would terminate in a salt-and-pepper mixture of differentiated cell types without any pattern.

Cell division and epigenetic mechanisms such as DNA methylation and histone modifications are the other crucial factors that influence the starting point and shape of the epigenetic landscape for each cell. To address these biological aspects, we suggest assigning individualized epigenetic landscapes to different cells, which are dynamically changed by the inherited parental cytoplasm and epigenetic modifications, the environmental signals and the other mechanisms of intercellular communication [34–38]. Hence the cells that are divided from the same parent or the adjacent cells would have similar epigenetic landscapes, which bias their differentiation towards particular cell types of the same tissue. We expect that this comprehensive approach to the Waddington landscape will provide new insights to the developmental biology.

## Conclusions

In this work we presented a framework for modeling the epigenetic landscape of the single cell resolution gene expression profiles. We visualized the epigenetic landscape of mouse preimplantation embryogenesis based on the expression profiles of 48 genes in 442 embryonic cells [8], which resembled the original metaphoric Waddington landscape of cellular differentiation [1]. Next we scrutinized to determine the regulatory circuitry that governs each developmental bifurcation.

We examined, through an ODE based model, the two-TF genetic circuits, which were previously suggested to regulate lineage bifurcations [5]. Perturbation, in form of increased decay rate of one TF, severely changed the shape and position of the attractor states. It could be concluded that any factor that has the potential to affect the expression or function of those TFs, such as genetic mutations, extrinsic stimuli and intrinsic noise, could deviate the corresponding cell fate decision.

Next we developed a hierarchical regulatory network consisting in pairs of auto-activating and mutual-inhibiting clusters of TFs. Our analysis showed the enhanced buffering capacity of the two-cluster regulatory circuitry against biological perturbations, due to the collective decision-making of TFs. Our finding can be a further explanation for the determinism and robustness of the embryonic development.

## Methods

We employed two different approaches to model the cell differentiation processes. In the first approach we used the experimental data to visualize the Waddington landscape of early mouse embryogenesis and identified the clusters of the genes differentially expressed in each developmental bifurcation. In the second approach, we theoretically compared the dynamical systems generated by the smaller (two-TF) and the extended (two-cluster) regulatory circuitries, using ODE based models.

### Waddington landscape: preprocessing of the experimental data

We obtained the expression profiles of 48 genes in 442 single mouse embryonic cells from zygote to 64 cells stage, that were generated by the TaqMan qRT-PCR assay [8]. These genes were selected after analyzing the expression levels of 802 TFs, due to differential expression in blastomeres or known function in early development. The initial Ct values ranged from 10 to 28, and the expression values were assigned by subtracting the Ct values from the baseline value of 28 (see the Additional file 3). PCA was performed using the mean-subtracted expression values. Correlation heatmap of the genes was generated based on pairwise Spearman correlations of the expression profiles of the cells in the 64-cell stage.

### Axes of Waddington landscape

In order to visualize the Waddington landscape of the preimplantation development, we needed to define each dimension and compute it. There are three axes (dimensions) in the epigenetic landscape, as illustrated in Fig. 1a: (i) The x-axis (left-right) through which distinct cell fates are shown as different attractors (valleys). (i) The y-axis (back-front) that shows time of development, as early and late developmental stages are located in backward forward of the landscape, respectively. (iii) The z-axis (down-up) that represents a potential function, which integrates both differentiation potency and stability [39]. The totipotent cells (zygotes) are posed at the highest valley. As the cells undergo more differentiation into pluripotent, multipotent and then unipotent cells, they go towards the deeper and lower valleys. Furthermore the stable cell states (attractors) are distinguished as valleys from the instable and transient cell states that form hills.

It was straightforward to assign the y-axis of the cells since the time of development was available for each cell. To establish the x-axis of the epigenetic landscape, we computed the principal components PC1 and PC2 of the gene expression profiles (Fig. 1b). The coordinates origin was slightly moved into a cell-free region (PC1 = −0.5, PC2 = 0) to ensure all the cells of the same fate are located in the same side of the origin and have close angular coordinates. Then the x-dimension of each cell was computed as its angular coordinate around the origin. Through this dimension reduction – from the initial gene expression profiles consisting of 48 dimensions into a single axis – we aimed to preserve the similarities and differences of the cells.

### Pseudo-potential function of the Waddington landscape

We needed to define a form of potential function from the experimental data. The closed form of a potential function is restricted to the gradient systems with stringent mathematical conditions that usually do not hold in biological systems [40]. As a result, most of the previous studies have defined pseudo- or quasi-potential functions based on many different methods: the ODEs with path integration [15, 40], Fokker-Planck equation [41, 42], Langevin dynamics [43], Hamilton-Jacobi equation [44], drift-diffusion models [45], Boltzmann distribution [46] and stochastic simulation [47]. Signaling network entropy, as a measure of promiscuity or undetermined lineage, is the other framework used to define a pseudo-potential function based on the experimental data [48].

In this study we employed the Boltzmann (Gibbs) distribution, which models the probability distribution of the particles in a system over various states with different energy levels [49]. It makes a connection between the energy levels and the probabilities of the particles being in each state. The Boltzmann distribution is expressed as the following equation:

$$ \frac{P(A)}{P(B)}={e}^{\raisebox{1ex}{$-\Delta E$}\!\left/ \!\raisebox{-1ex}{${k}_BT$}\right.} $$

where *A* and *B* are two different states, *P*(*x*) is the probability of a particle to be in state *x, ΔE* is the energy difference that a particle should absorb/release to change its state from *A* to *B*, *k*_*B*_ is the Boltzmann constant, and *T* is the system temperature. By taking the logarithm of two sides we have:

$$ \ln \frac{P(A)}{P(B)}=\frac{-\Delta E}{k_BT}\ \to\ E(A)-E(B)=-{k}_BT\left( \ln \left(P(A)\right)- \ln \left(P(B)\right)\right) $$

in which *E*(x) is the energy of a particle in state x. Taking the state *B* as the pseudo-potential reference results in:

$$ U\left(\mathrm{A}\right)=-\uprho\ \ln \left(P(A)\right)+\omega $$

where *U* is the pseudo-potential function. Both ρ and *ω* are constant values that scale the landscape and can be omitted in visualization. To compute the pseudo-potential function we should determine the probability of the cells to be in each state, as follows.

### Probability distribution of the cell states

At each developmental stage we assumed the expression profiles of the cells of the same type were normally distributed along the x-axis after the dimension reduction. To check this assumption we produced the Q-Q plots of each developmental stage for the angular coordinates of the cells in the PC1-PC2 plane (see the Additional file 4). Up to the 16-cell stage the points are almost fitting a single trend line. In the 32-cell stage there are two distinguished segments, discriminating ICM and TE cells. Each of three segments in 64-cell stage fit a different trend line, which shows this stage is a mixture of three normal distributions, representing EPI, PE and TE lineages. Furthermore we performed the Shapiro-Wilk normality test [50], that confirmed the normality of several segments of different stages.

As a result we considered a cell population including *m* different cell types would have a mixture of *m* normal distributions. By assuming *τ*_*k*_ as the probability of a cell belonging to the *k* -th cell type ($1\le k\le m,\ {\tau}_k\ge 0,\ {\displaystyle \sum_{k=1}^m}{\tau}_k=1$), the mixed probability distribution function is:

$$ f(x)={\displaystyle \sum_{k=1}^m}{\tau}_k{\Phi}_k\left(x\Big|{\mu}_k,{\Sigma}_k\right) $$

where *μ*_*k*_ and Σ_*k*_ are the mean and covariance matrix of all the cells of the *k* -th cell type, and Φ_*k*_ is a Gaussian function defined as:

$$ {\Phi}_k\left({\boldsymbol{x}}_{\boldsymbol{i}}\Big|{\mu}_k,{\Sigma}_k\right)=\frac{1}{\sqrt{2\pi \left|{\Sigma}_k\right|}}{e}^{-\frac{1}{2}{\left({\boldsymbol{x}}_i-{\mu}_k\right)}^T{\Sigma}_k^{-1}\left({\boldsymbol{x}}_i-{\mu}_k\right)} $$

From the above equations we could calculate the pseudo-potential function:

$$ U(x) \propto - \ln \left(f(x)\right) $$

where *x* is any point on the x-axis of the epigenetic landscape (projection of the gene expression profiles) at some particular developmental time. The mixed distribution and the pseudo-potential function were recalculated for each developmental stage with the available experimental data. A linear interpolation was used to fill the gaps between consecutive developmental stages. The landscape was visually tilted to show the reduced differentiation potency during development.

Selecting the number of different cell types and assigning each cell to one of cell types can be done either manually (supervised) or computationally (unsupervised). To have an objective and automated framework, we used the unsupervised approach, using the “mclust” package [51] of R statistical language. The projected expression profiles were given to the package to compute the probabilistic model parameters, including the number of cell types (clusters), the mean and covariance values, based on a maximum likelihood criterion. For additional details, one may refer to the “mclust” package reference manual.

### Dynamical modeling: Phase space representation of the regulatory circuitry of two transcription factors (TFs)

For the dynamical system analysis of the two-TF regulatory circuitry, we employed the following set of ODEs [3, 39]:

$$ \frac{du}{dt}=\alpha \frac{u^n}{s^n+{u}^n}+\beta \frac{s^n}{s^n+{v}^n}-\gamma u $$

$$ \frac{dv}{dt}=\alpha \frac{v^n}{s^n+{v}^n}+\beta \frac{s^n}{s^n+{u}^n}-\gamma v $$

where *u* and *v* are concentrations of the pair of opposite TFs, and *α* and *β* are the strengths of the positive and negative regulatory interactions, respectively. For simplicity we used the same protein degradation rate *γ* for both TFs. The term $\frac{x^n}{s^n+{x}^n}\ \left(x\in \left\{u,v\right\}\right)$ is a sigmoid function that has 0 value at *x* = 0, increases to 0.5 at *x* = *s*, and asymptotically approaches 1 at the large values of *x*. It resembles the positive auto-activation regulatory effect of each TF. The steepness of the sigmoid function is defined by the power *n*. On the other hand, $\frac{s^n}{s^n+{x}^n}$ is a decreasing sigmoid function that starts from 1 at *x* = 0 and approaches 0 at large *x* values, which resemble the mutual inhibitory effects.

In order to model the perturbation in the form of increased decay rate of a particular protein, we increased the degradation rate of the TF *u* by 50 %, as denoted by γ*:

$$ \frac{d{u}^{*}}{dt}=\alpha \frac{u^n}{s^n+{u}^n}+\beta \frac{s^n}{s^n+{v}^n}-{\gamma}^{*}u $$

### Phase space representation of the regulatory circuitry of two clusters of transcription factors (TFs)

To analyze the dynamical system of a gene regulatory circuitry consisting in two clusters of TFs we generalized the previous two-TF model by using the following equations:

$$ \frac{dx}{dt}=\frac{d{u}_1}{dt}+\frac{d{u}_2}{dt}=\eta \left({u}_1,{u}_2,{v}_1,{v}_2,\gamma \right)+\eta \left({u}_2,{u}_1,{v}_2,{v}_1,\gamma \right) $$

$$ \frac{dy}{dt}=\frac{d{v}_1}{dt}+\frac{d{v}_2}{dt}=\eta \left({v}_1,{v}_2,{u}_1,{u}_2,\gamma \right)+\eta \left({v}_2,{v}_1,{u}_2,{u}_1,\gamma \right) $$

where *u*_1_ and *u*_2_ are the concentrations of two proteins of the first cluster, *v*_1_ and *v*_2_ denote the second cluster protein concentrations, and *x* = *u*_1_ + *u*_2_ and *y* = *v*_1_ + *v*_2_ are the total concentrations of the proteins in clusters 1 and 2, respectively. We defined the generic function *η*(*a*, *b*, *c*, *d*, *γ*) to compute the concentration rate of any protein *a* based on the concentration values of the TFs *a* and *b* in one cluster, and *c* and *d* in the other cluster, as follows:

$$ \eta \left(a,b,c,d,\gamma \right)=\alpha \frac{a^n+{b}^n}{s^n+{a}^n+{b}^n}+\beta \frac{s^n}{s^n+{c}^n+{d}^n}-\gamma a $$

For the perturbation analysis, we used the increased degradation rate γ* for the TF *u*_2_:

$$ \frac{dx}{dt}=\frac{d{u}_1}{dt}+\frac{d{u}_2^{*}}{dt}=\eta \left({u}_1,{u}_2,{v}_1,{v}_2,\gamma \right)+\eta \left({u}_2,{u}_1,{v}_2,{v}_1,{\gamma}^{*}\right) $$

In both models we used the following parameters: *n* = 4, *s* = 0.5, *α* = 1.5, *β* = 1, *γ* = 1 and γ* = 1.5. The sample space of (*u*, *v*) = [0, 3]^2^ was used for analyzing the two-TF model, and (*u*, *v*, *u*_1_, *v*_1_) = [0, 3]^4^ for the two-cluster model.

### Simulation of the cell differentiation in absence or presence of perturbation

For each of the four different regulatory circuitries depicted in Fig. 3c, d and Fig. 4a, b we simulated the differentiation of 1000 cells. The initial expression rates of the TFs in each cell were assigned from a normal distribution with *μ* = 1.5 and *sd* = 1. With this selection of parameters, the majority of the cells were initially in proximity of the progenitor state (the attractor 2 of Figs. 3 and 4).

Each simulation continued 100 steps, in which the expression rates of the TFs in each cell were slightly changed, based on two factors: the dynamical system forces (differential equations above) and a standard Gaussian noise (*μ* = 0, *sd* = 1). The strengths of these factors were tuned by two coefficients: the force field coefficient had a constant value of 0.2 during the simulation; and the noise coefficient that started with 0.5 and gradually reduced during the simulation (multiplied by 0.98 in each step) to ensure the convergence of the experiment.

The auto-activation strength *α* was 1.5 at the beginning of each simulation, but gradually reduced (multiplied by 0.98 in each step). In this way, we forced the cells to leave the progenitor state 2 and differentiate into the attractor states 1 or 3. During this process, the stability of the attractor 2 was gradually decreased and resulted in a bistable system with only attractors 1 and 3. In each attractor of the bistable system, one TF was silent and the other was expressed at a slightly lower rate than the initial circuit configuration, due to the lower value of *α*.

### Implementation

The code was implemented in R statistical language [52]. We used the packages “mclust” to generate the mixed Gaussian model, “rgl” for 3D visualization of the Waddington landscape, “ggplot2” for 2D visualization of the data [53], and “pheatmap” for visualization of the correlation heatmap. We also used the packages “grid”, “gplots”, “plyr”, “Hmisc”, and “Biobase”.

### Advantages and limitations

Our method of visualizing the Waddington landscape enables the application of the experimental data at single cell resolution for this purpose. While we used the gene expression profiles of early embryonic cells, our method can be generalized for analysis of the high-throughput DNA methylation, histone modifications and non-coding RNA expression profiles. It is computationally fast and can be used for whole-genome scale of data and a large number of single cells. By application of time-course data, the same method can be applied for visualizing the landscape of reprogramming, transdifferentiation or stem cell differentiation.

Our method interpolates the developmental time between each pair of successive sampling time points; hence the closer the sampling time points, the more realistic the resulting landscape. The valley depth in this method mainly represents the number of cells assigned to the corresponding attractor state. This requires the data to be generated by random sampling of different cell types. For study of distant cell types, the quantity of cells and the depth of attractors can be influenced by cell division rates. In this case we suggest combining our method with an indicator of differentiation potency or stability, such as the cellular network entropy [48].

### Availability of supporting data

The preprocessed single-cell resolution gene expression profiles of mouse preimplantation embryonic cells [8] are provided in the Additional file 3. We have also provided in the same additional file the complete source code of this study in R programming language.

## Additional files

Additional file 1:
**The simulation results of the differentiation of 1000 cells with two-TF (plots 1 and 2) or two-cluster regulatory circuitries (plots 3 and 4).**
Additional file 2:
**The expression profiles of the TF clusters in the embryonic cells.** We computed the average expression levels of the TFs of each cluster in each cell, and colored the cell accordingly. The cells with the highest expression level of each cluster are depicted in red, while the intermediate and the lowest expression levels are shown in white and blue, respectively. Three TF clusters responsible for EPI, PE and TE differentiation are shown. Additional file 3:
**The complete source code of the study, in R programming language, and the preprocessed data.**
Additional file 4:
**The Q-Q plots of the angular coordinates of the gene expression profiles in the (PC1, PC2) plane.**

## Abbreviations

TF: Transcription factor
ODE: Ordinary differential equation
ICM: Inner cell mass
TE: Trophectoderm
EPI: Epiblast
PE: Primitive endoderm
PCA: Principal component analysis
PC: Principal component
BIC: Bayesian information criterion
qRT-PCR: Quantitative reverse transcription polymerase chain reaction
Ct: Cycle threshold.
