# Supplementary material for: Increased robustness of early embryogenesis through collective decision-making by key transcription factors
Source: BMC Syst Biol. 2015 Jun 2;9:23. doi: 10.1186/s12918-015-0169-8 (PMC4450992; doi:10.1186/s12918-015-0169-8)
Supplement: Additional file 1: — The simulation results of the differentiation of 1000 cells with two-TF (plots 1 and 2) or two-cluster regulatory circuitries (plots 3 and 4). [file 12918_2015_169_MOESM1_ESM.pdf]

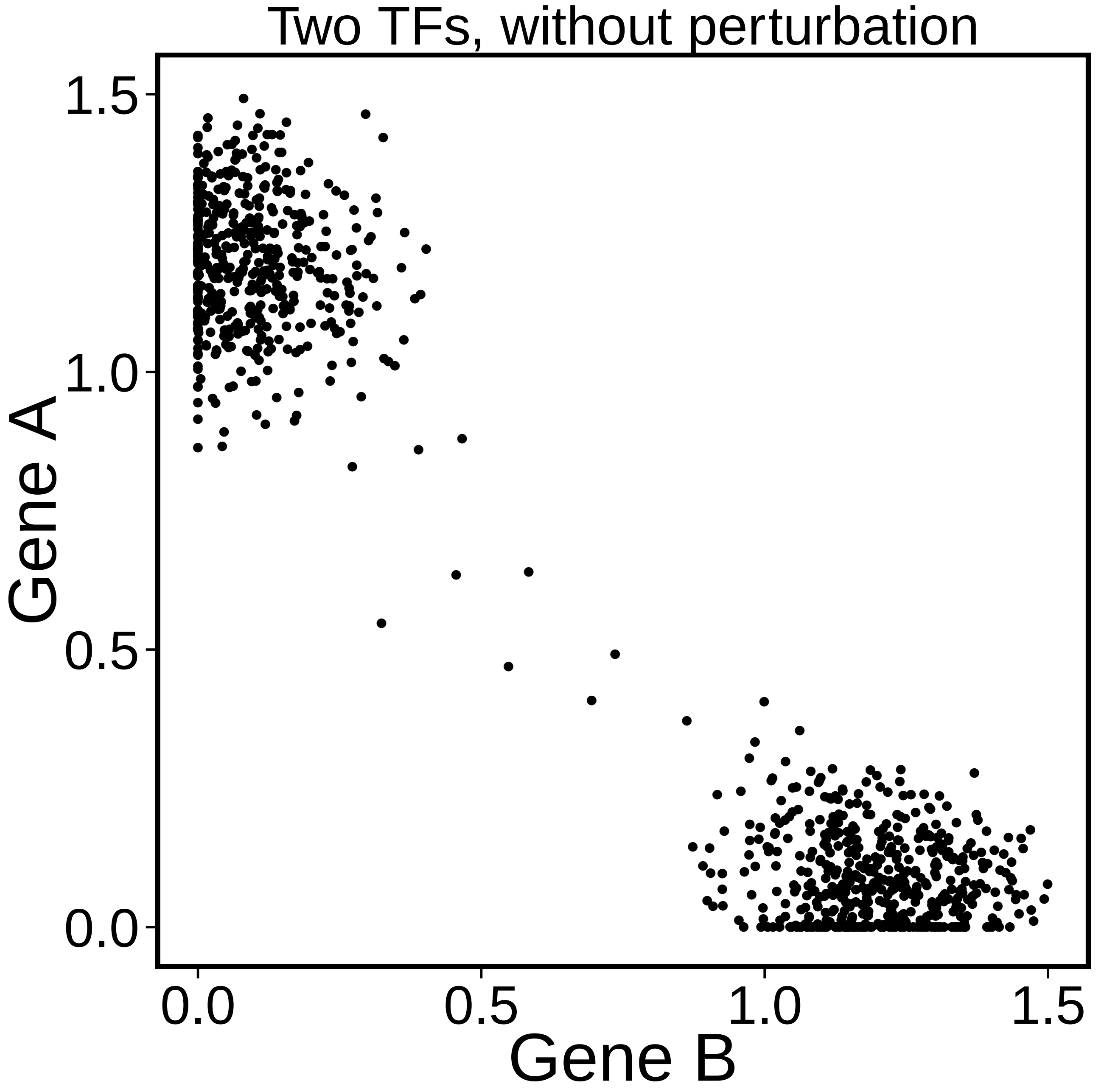
Two TFs, without perturbation
Gene A
Gene B
0.0
0.5
1.0
1.5

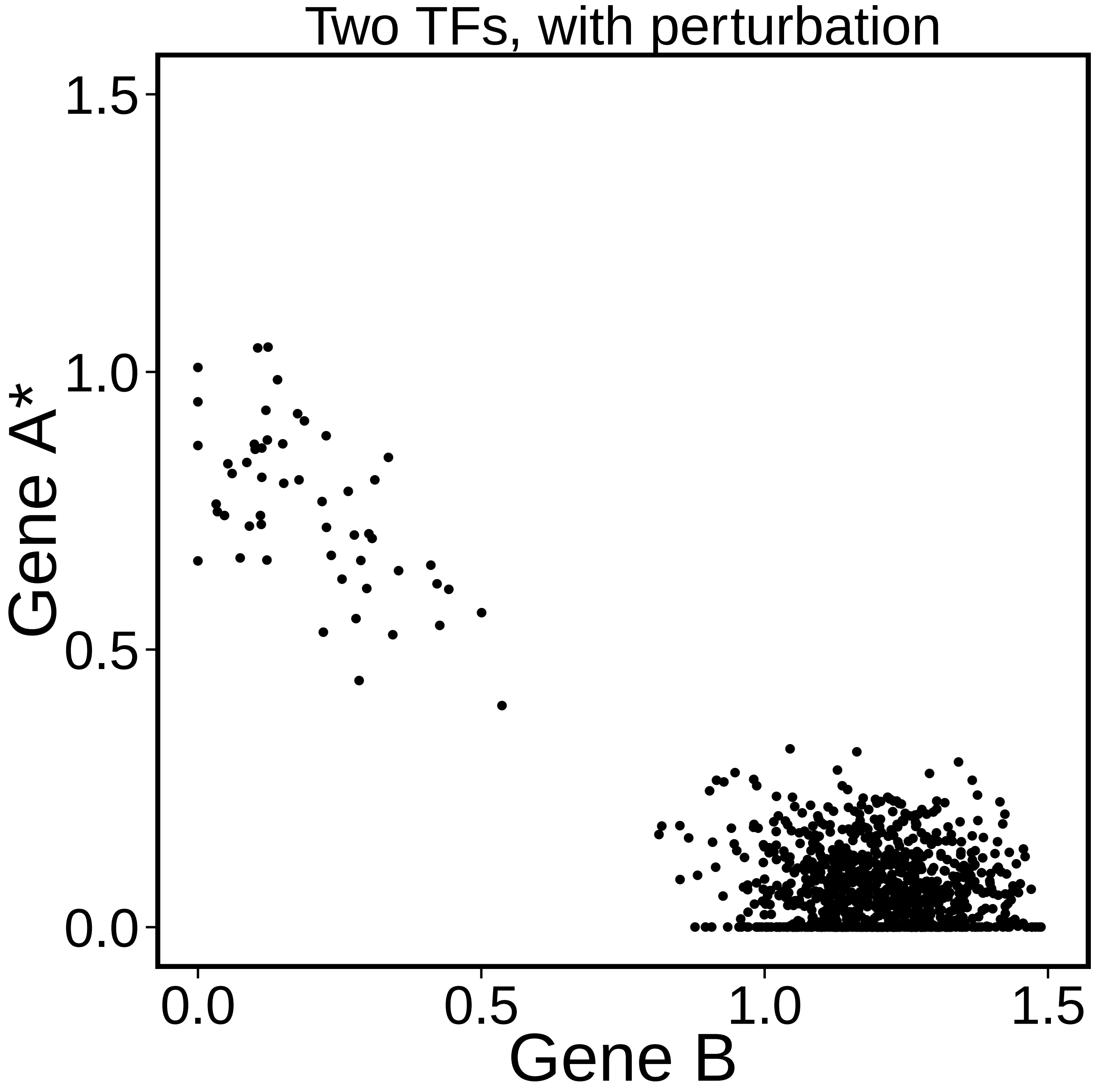
Two TFs, with perturbation
Gene A*
Gene B
1.5
1.0
0.5
0.0
0.0
0.5
1.0
1.5

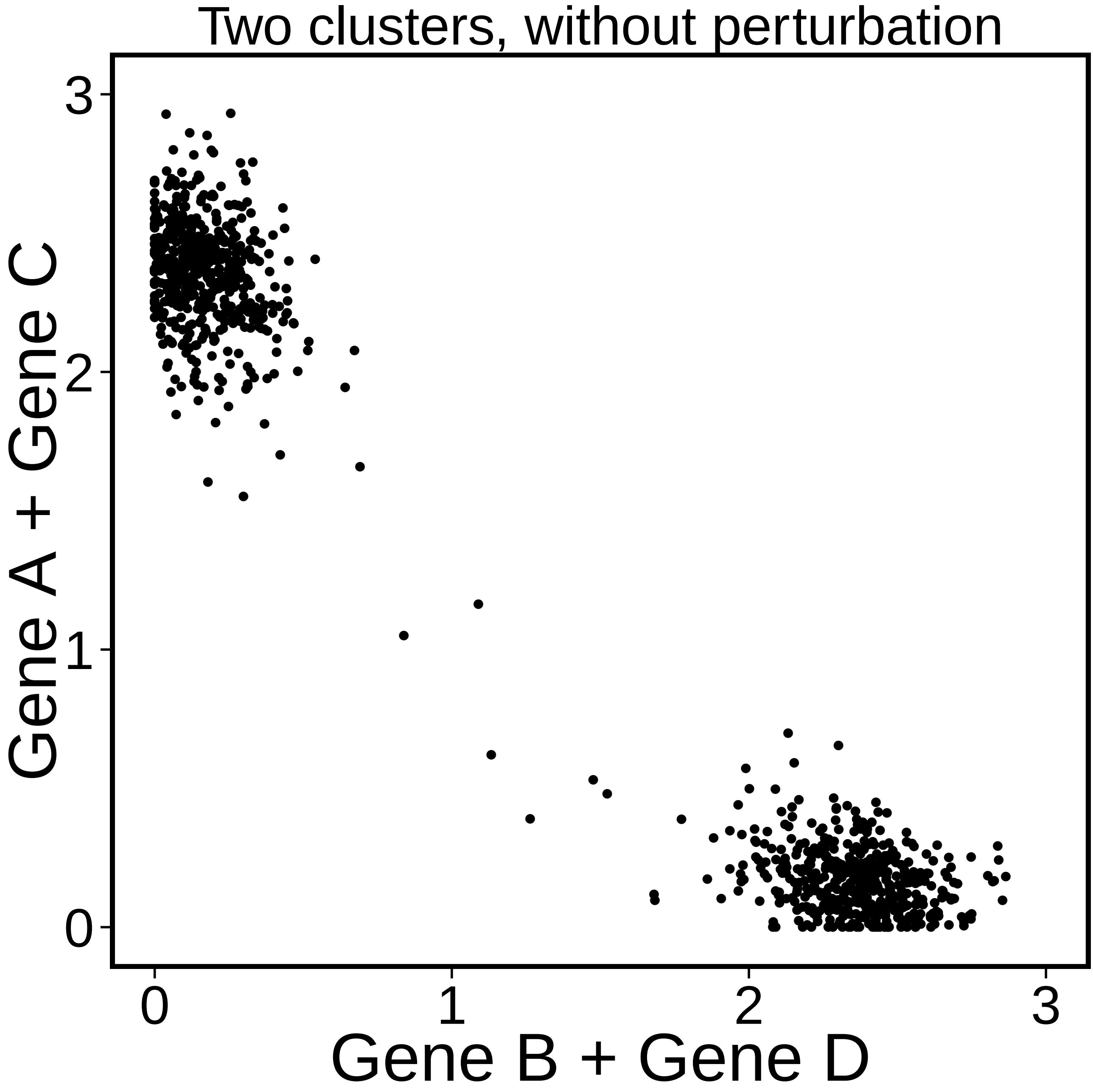
Two clusters, without perturbation
Gene A + Gene C
Gene B + Gene D
0
1
2
3
0
1
2
3

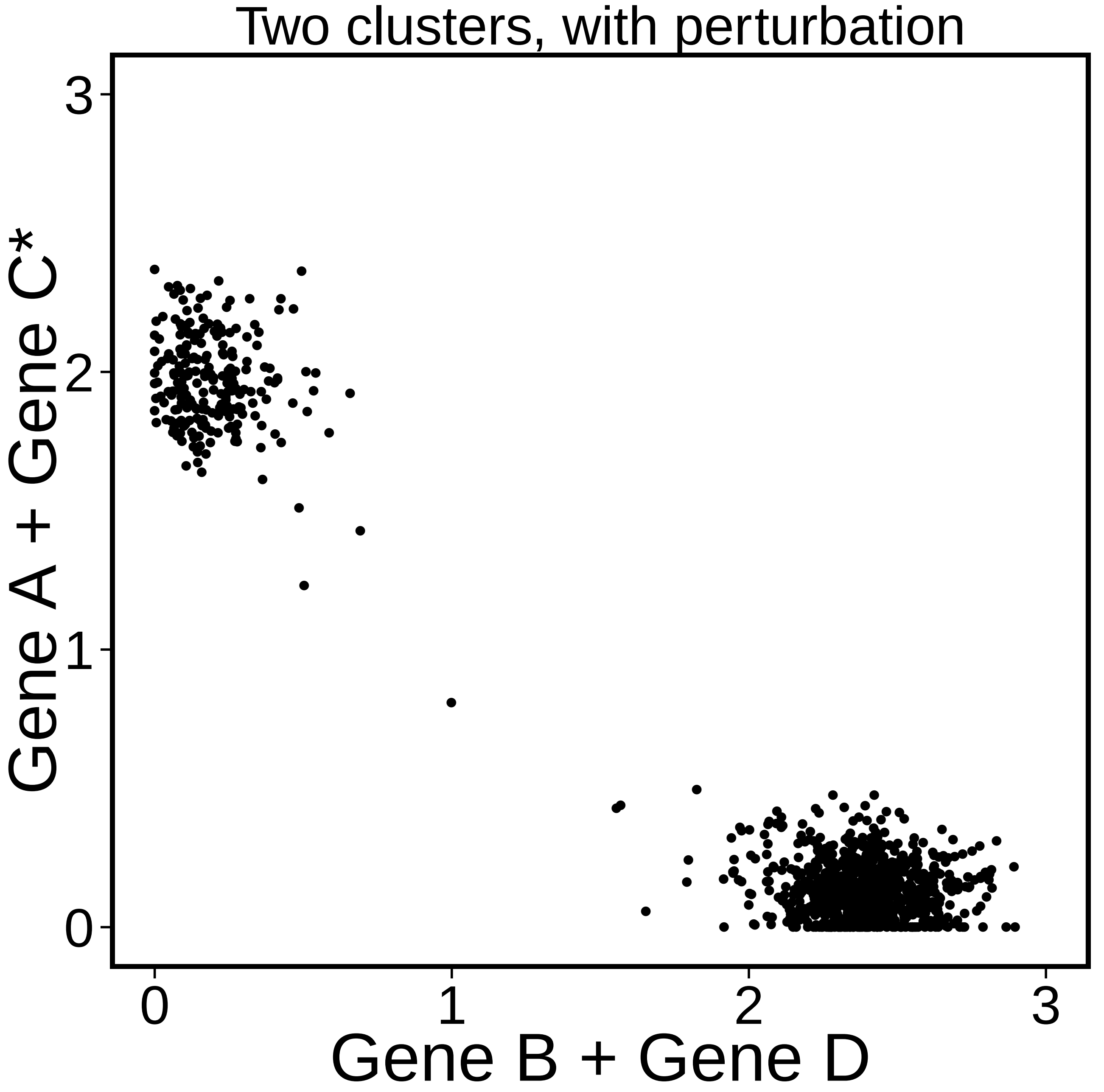
Two clusters, with perturbation
Gene A + Gene C*
Gene B + Gene D
0
1
2
3
0
1
2
3
