# Supplementary figures and images for: Increased robustness of early embryogenesis through collective decision-making by key transcription factors
Source: BMC Syst Biol. 2015 Jun 2;9:23. doi: 10.1186/s12918-015-0169-8 (PMC4450992; doi:10.1186/s12918-015-0169-8)

EPI Cluster  
Nanog, Oct4, Sox2

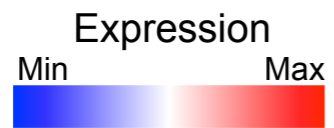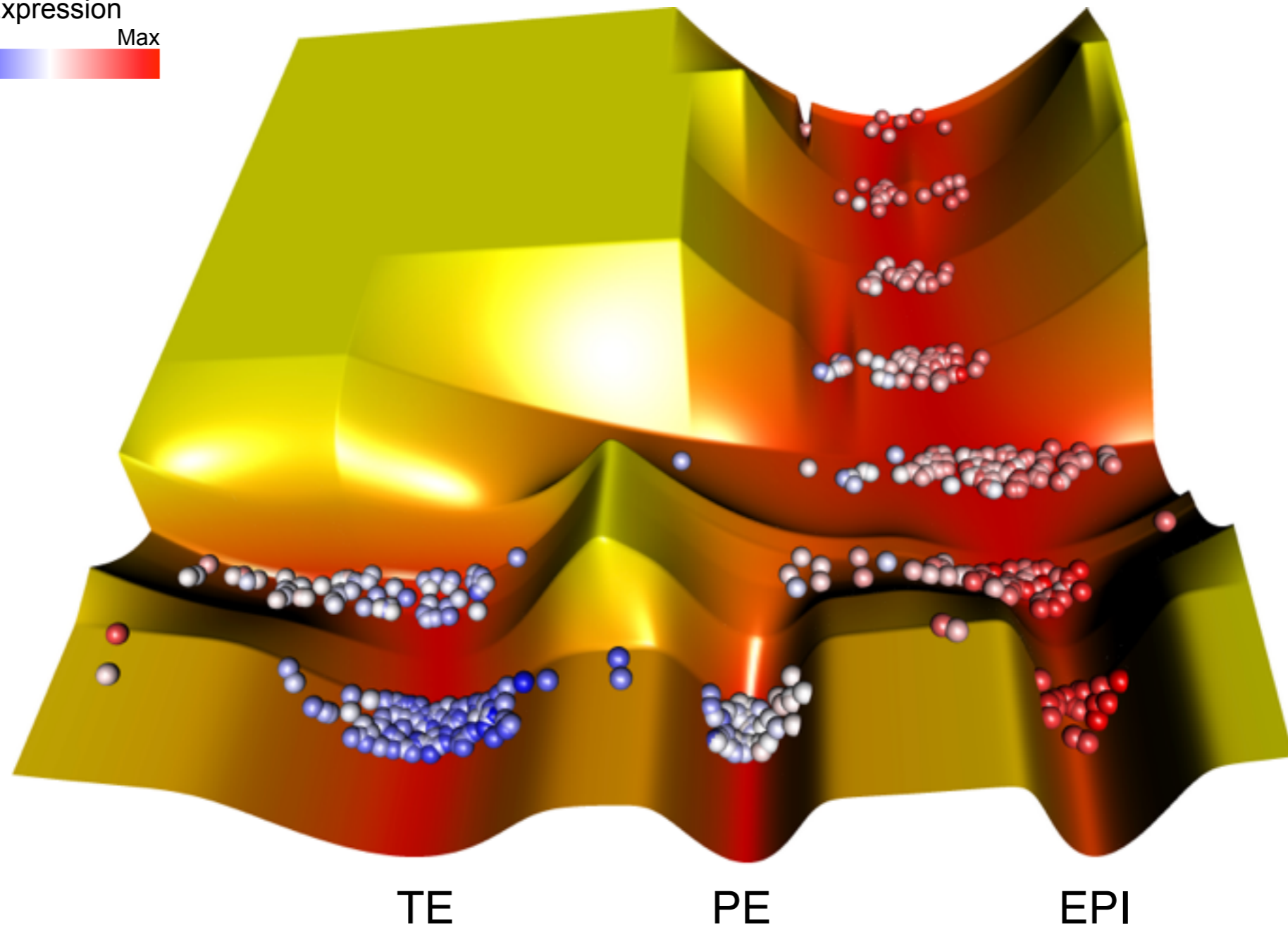

PE Cluster  
Gata6, Sox17, Gata4

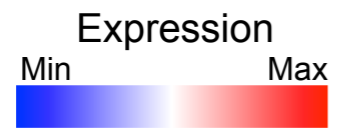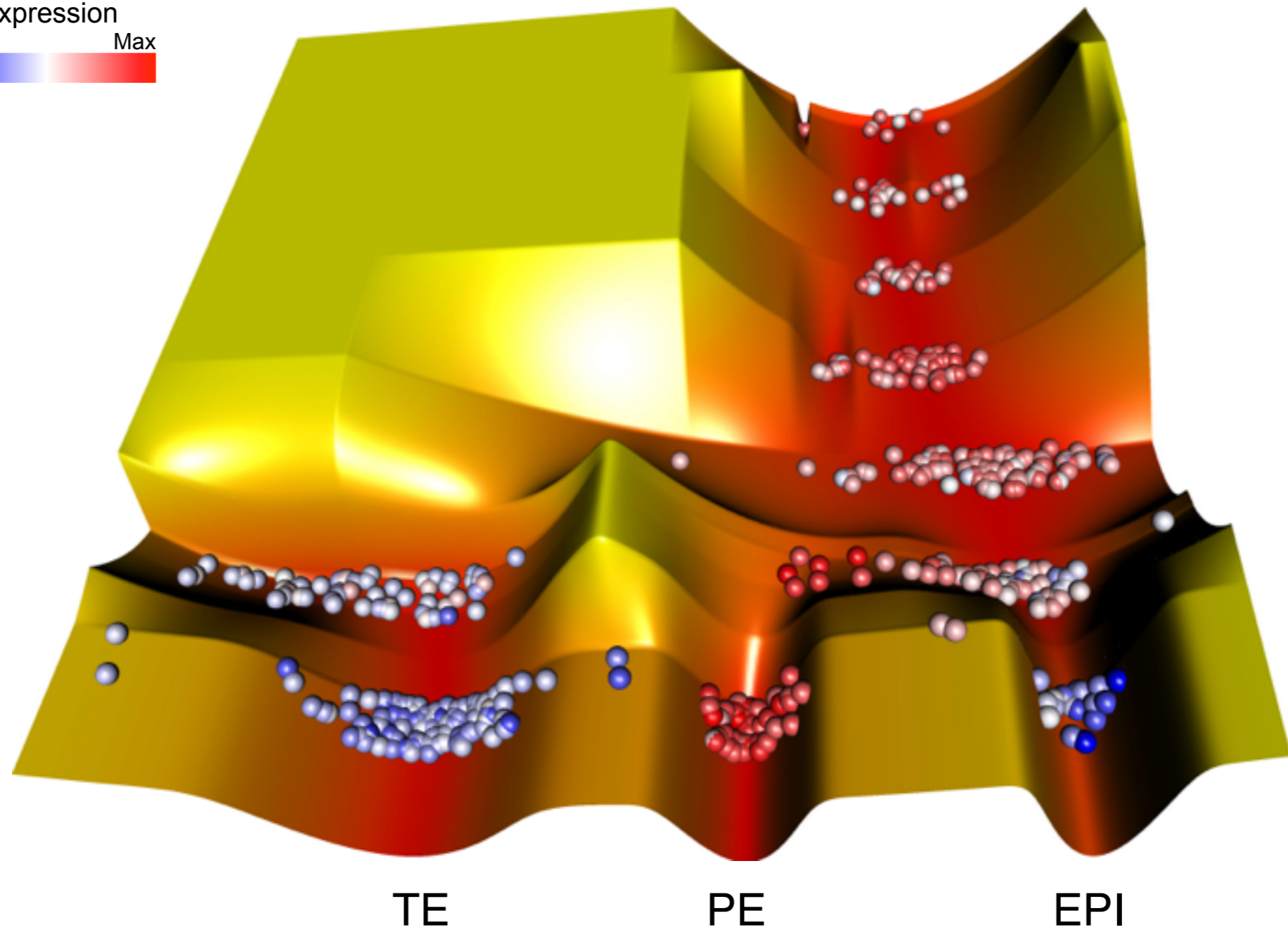

TE Cluster  
Cdx2, Gata3, Eomes

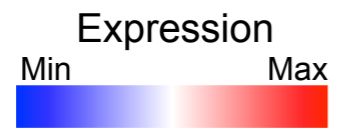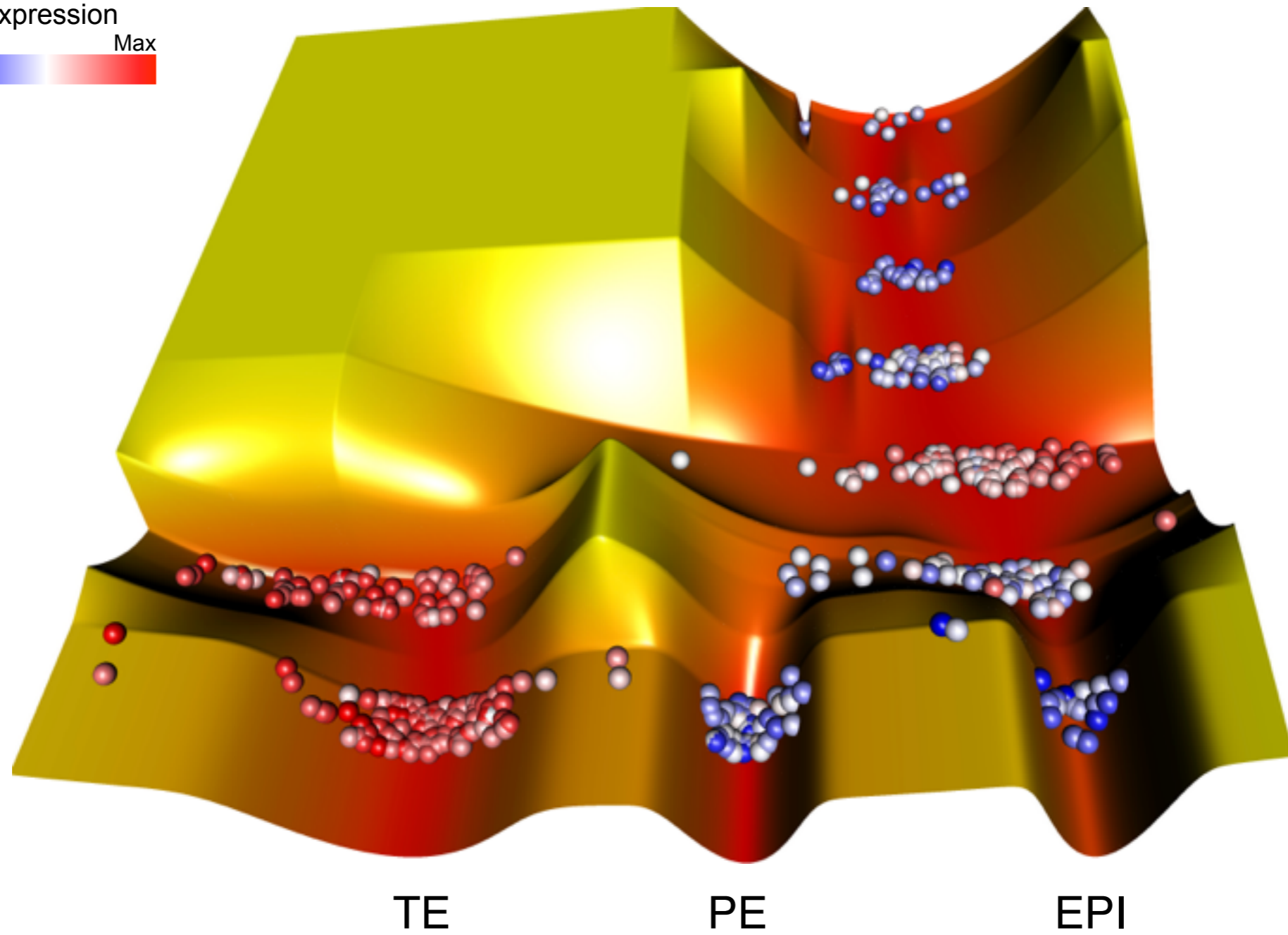

Supplement: Additional file 2: — The expression profiles of the TF clusters in the embryonic cells. We computed the average expression levels of the TFs of each cluster in each cell, and colored the cell accordingly. The cells with the highest expression level of each cluster are depicted in red, while the intermediate and the lowest expression levels are shown in white and blue, respectively. Three TF clusters responsible for EPI, PE and TE differentiation are shown. [file 12918_2015_169_MOESM2_ESM.pdf]

**Q-Q Plot for the 1-cell to 16-cell stages**

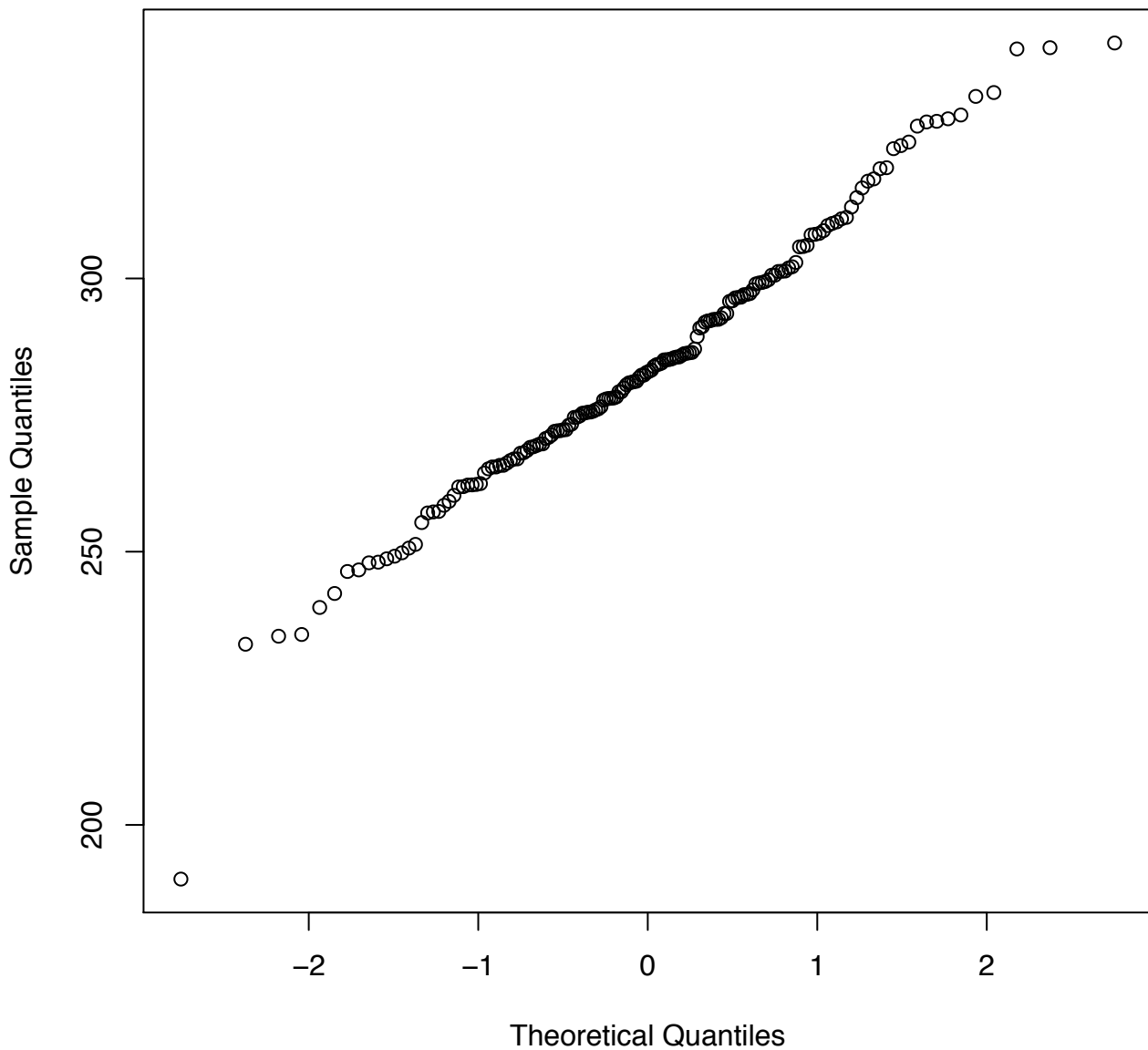

**Q-Q Plot for the 32-cell stage**

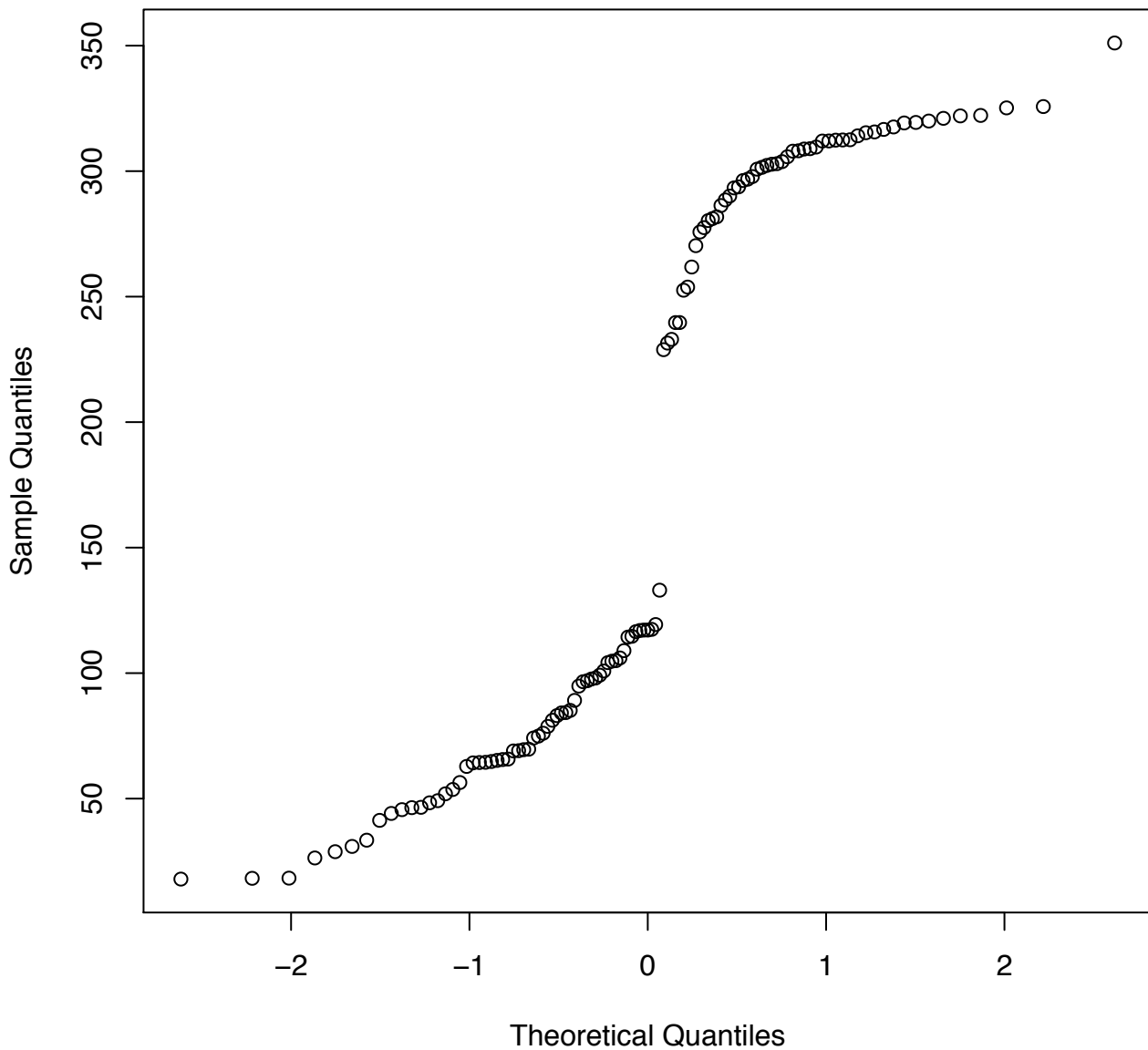

**Q-Q Plot for the 64-cell stage**

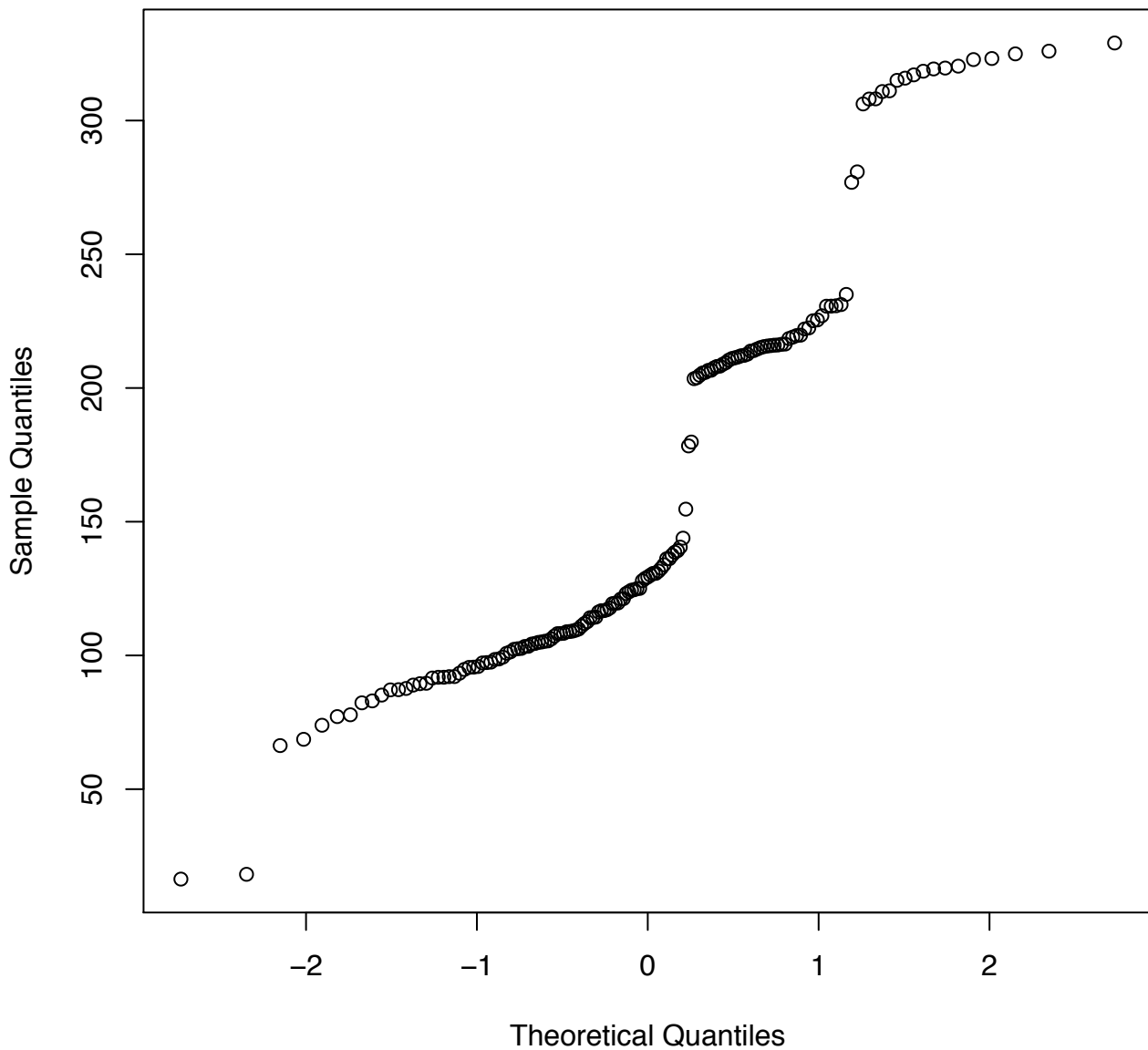

Supplement: Additional file 4: — The Q-Q plots of the angular coordinates of the gene expression profiles in the (PC1, PC2) plane. [file 12918_2015_169_MOESM4_ESM.pdf]
