# Supplementary material for: Increased robustness of early embryogenesis through collective decision-making by key transcription factors
Source: BMC Syst Biol. 2015 Jun 2;9:23. doi: 10.1186/s12918-015-0169-8 (PMC4450992; doi:10.1186/s12918-015-0169-8)
Supplement: Additional file 3: — The complete source code of the study, in R programming language, and the preprocessed data. [file 12918_2015_169_MOESM3_ESM.zip › Additional file 4/Read Me.pdf]

The preprocessed data

Preprocessed expression profiles of 48 genes in 442 single cells of the mouse early (zygote to 64-cell stage) embryogenesis. Rows and columns represent single cells and genes, respectively. Expression values range from 0 (no expression) to 17.96 (maximum expression). The original data is obtained from [1]. The data is stored in "Data/Data.txt"

The source codes

The source codes are available in the "Source" folder. Here are the requirements for running the source code:

- R programming language, available on www.r-project.org
- (optional) RStudio, the IDE for R, available on www.rstudio.com
- The R packages: mclust, rgl, Biobase, ggplot2, gplots, grid, Hmisc, pheatmap and plyr. These packages can be obtained from CRAN and Bioconductor package repositories.
- To run rgl package you might need to install X11, depending your operating system. The X11 for mac is available on http://xquartz.macosforge.org/trac

Generating PDF files of the Waddington landscapes might take a few minutes, depending your system performance.

There is also a folder "GraphicalSettings" containing the graphical parameters (including the view angle) for rendering the 3D landscapes.

Results

After successful running of the source codes, the results will be stored in "Results" folder. This folder is currently empty.

Citation

This code is freely available, AS-IS. We appreciate citing the article whenever you used the code in any research.

References

1. Guo G, Huss M, Tong GQ, Wang C, Sun LL, Clarke ND, Robson P: **Resolution of Cell Fate Decisions Revealed by Single-Cell Gene Expression Analysis from Zygote to Blastocyst**. *Developmental Cell* 2010, **18**:675–685.
